# Updates in Management of Acute Disorders of Consciousness After Traumatic Injury

**DOI:** 10.3390/brainsci16060613

**Published:** 2026-06-04

**Authors:** Taylor S. Hudson, Brianne M. Seagreaves, Amelia W. Maiga

**Affiliations:** 1 Section of Surgical Sciences, Vanderbilt University Medical Center, 1161 21st Avenue South, Medical Center North, Nashville, TN 37232, USA; taylor.scott.hudson@vumc.org; 2 Critical Illness, Brain Dysfunction, and Survivorship (CIBS), Center for Health Services Research, Nashville, TN 37203, USA; brianne.seagreaves@vumc.org; 3 Division of Acute Care Surgery, Vanderbilt University Medical Center, 1211 21st Avenue South Suite 404, Medical Center North, Nashville, TN 37232, USA

**Keywords:** disorders of consciousness, brain injury, consciousness, coma, trauma

## Abstract

**Highlights:**

**What are the main findings?**

- Prognosticating for a suspected disorder of consciousness is challenging immediately after traumatic injury.
- Diagnosing a suspected disorder of consciousness is possible in the ICU.

**What are the implications of the main findings?**

- Early prognostic anchoring should be avoided after traumatic injury with a suspected disorder of consciousness.
- The CRSR-FAST should be used in the ICU to evaluate for suspected disorders of consciousness.

**Abstract:**

Traumatic injury is one of the most common causes of disorders of consciousness (DoC) worldwide, but the management and prognosis of DoC remain enigmatic. The uncertainty surrounding the natural course of DoC, the tendency of consciousness to wax and wane, and a lack of effective treatments outside of avoiding additional insults renders trauma-associated DoC complex for both providers and patient surrogates to navigate. This review explores the acute clinical course of DoC after traumatic injury chronologically and aims to compile recommendations based on the current best practices for diagnosis, management, and prognostication when caring for these patients during their acute hospitalization. Updates from trauma and Traumatic Brain Injury (TBI) resources, such as the American College of Surgeons, as well as new recommendations in the field of DoC are summarized. Serial clinical assessment with a standardized neurobehavioral battery such as the CRSR-FAST remains the mainstay of clinical care and research for DoC. Accurate diagnosis, multifaceted management, and humility surrounding prognostic discussions are all critical to caring for patients with DoC after trauma. Most of the care for trauma patients with a DoC remains supportive and aimed at avoiding secondary insults while allowing time for the patient’s recovery. In the same way that clinical care focuses on a cycle of diagnosis, treatment, and prognosis with each providing insight for the next, ongoing and future DoC research will compound on itself and hopefully lead to more advances in the future.

## 1. Introduction

Traumatic injury resulting in acute brain injury and disorders of consciousness (DoC) are frequently coinciding pathologies in the acute care setting. Even traumatic brain injuries traditionally classified as mild have the potential to lead to at least a brief period of decreased consciousness or post traumatic amnesia [1,2,3,4]. A DoC is characterized by absent or fluctuating wakefulness or awareness [5]. The four major DoC states (coma, vegetative state/unresponsive wakefulness syndrome, minimally conscious state, and post-traumatic confusional state), are well defined, but can be difficult to diagnose and differentiate, particularly in the chaos of the acute setting [5,6]. While some patients have early resolution of their DoC, others have a more prolonged course, into the post-acute rehabilitation setting. For some, the post-acute setting may be the first time they will be labelled as having a DoC, despite meeting the criteria for weeks in the acute setting. Many aspects of the diagnosis, management, and prognosis of DoC still need additional exploration, which is particularly difficult given the heterogeneity and spectrum of brain injury leading to DoC.

Acute brain injury in the setting of trauma is both a common and complex problem. It is estimated that 4.8 million ED visits, 214,000 hospitalizations, and 69,000 mortalities per year in the United States are associated with traumatic brain injury (TBI) alone [7,8]. Primary TBI is not the only cause of brain injury in the setting of a traumatic injury. Other etiologies include hypoxic or anoxic brain injury (often occurring peri-arrest due to polytrauma associated with hemorrhagic shock), fat emboli, and the sequelae of critical illness after traumatic injury [9,10]. These other etiologies can also result in clinically significant brain injury in a trauma patient as either a primary acute brain injury themselves, or as a “secondary hit” in the setting of structural neurotrauma. Nevertheless, TBI accounts for most of the acute brain injury and DoC in trauma patients and has the highest incidence and prevalence of all neurological disorders [11].

TBI frequently involves a DoC [12]. Primary TBI is heterogeneous in nature, and secondary insults are often more harmful than the primary traumatic insult. For example, hypotensive episodes following trauma are associated with higher severity and mortality, and the development of acute respiratory distress is an independent predictor of mortality in hypotensive trauma patients [13,14]. For these reasons, trauma patients with brain injury must be managed with consideration of not just the brain injury, but of all injuries and pathologies tied to the traumatic event and acute hospitalization. Optimal care of acute DoC means optimal care of the whole patient. The updated 2024 guidelines from the American College of Surgeons (ACS) regarding management of traumatic brain injury provide best practice recommendations for TBI with several updates from the previous 2015 recommendations [15,16]. These include updates in the areas of neuroimaging, blood-based biomarkers, seizure prophylaxis medications, intracranial pressure management, and rehabilitation [15,16]. While these guidelines only briefly address disorders of consciousness by name, the theme of DoC and the importance of recognition, diagnosis, treatment, and prognosis of patients with decreased levels of consciousness is emphasized throughout. The care of neurotrauma patients, particularly those with DoC, requires a multidisciplinary team capable of diagnosing and managing patients from the time of injury, during acute care, and throughout post-acute care and recovery. Trauma is one of the most common causes of DoC worldwide, and this review aims to compile recommendations based on the current best practices and updates for DoC [17].

## 2. The Acute Care Context

### 2.1. Hyper-Acute Window—The First 24 h: Emergency Room/Trauma Bay

**Figure 1:**
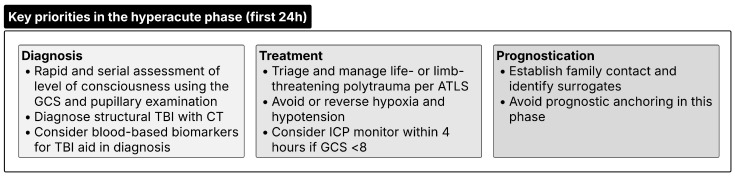
Key priorities in the hyperacute phase (first 24 h). This figure summarizes the key priorities for patients with suspected disorders of consciousness in the first 24 h after injury. GCS, Glasgow Coma Scale; TBI, traumatic brain injury; CT, computed tomography; ATLS, Advanced Trauma Life Support; ICP, intracranial pressure.

An impaired sensorium is common after trauma, and the etiology is often mixed. In a review of 1.6 million patients who sustained blunt trauma, 39.1% had TBI, 3.7% had moderate to severe TBI, and 9.1% had a prehospital Glasgow Coma Scale (GCS) of ≤12 [18]. The first several minutes-to-hours of the assessment and management of these patients is one of the most critical periods of their medical care. For this reason, we often refer to the “golden hour” in trauma care [19]. Early management addressing both the suspected brain injury and any additional traumatic injuries begins in transport, before the patient has even reached the hospital. This management includes a focus on avoiding or reversing hypoxia and hypotension. Rapid and serial assessment of the level of consciousness is of utmost clinical relevance in this hyper-acute phase as it drives important decisions about approaches to care, triage location, and early prognostic anchoring [16,20]. The key priorities in the hyperacute phase are summarized in Figure 1.

#### Diagnosing and Managing DoC in the Hyper-Acute Window

Diagnosis and management occur simultaneously in the first 24 h. TBI is typically a highly heterogeneous, multifocal injury pattern with focal brain damage and/or diffuse axonal injury, and recovery of consciousness and other brain functions is variable and occurs over time through reemergence of dynamic cortical and subcortical networks [17]. The bedside exam remains the gold standard for assessment of consciousness in acute trauma. Clinical assessment of neurological status is most often performed using the GCS, as it is a well-known, efficient, and validated test that can be performed quickly and repeatedly at bedside. In addition to the total sum score, component scores of the GCS should be documented. Trauma patients with a total GCS less than 13 or a GCS motor component subscore less than 6 are judged as probable to have a TBI, and thus ACS guidelines recommend rapid transport of these patients to the highest-level trauma center available, along with corrective actions for vital sign and lab abnormalities en route, particularly treatment to avoid hypotension and hypoxia [16]. In this way, management of TBI/DoC has already begun prior to any formal diagnosis of brain injury or DoC.

Once the patient arrives at the hospital, diagnosis and management continue in parallel, most commonly using the Advanced Trauma Life Support (ATLS) treatment algorithms [21]. Triage of injuries, and prioritized management of those injuries, occurs immediately on arrival (referred to as “x-ABCDE” in ATLS, which stands for exsanguinating external hemorrhage, airway, breathing, circulation, disability/neurologic assessment, and exposure), with plain radiographic films and bedside ultrasound intercalated as diagnostic adjuncts for cavitary triage [21]. The disability or neurologic assessment can be confounded by many factors beyond TBI, such as sedation, pain medication, intoxication, post-ictal state, hypotension, and polytrauma. Additionally, pupillary assessment with documented size and light reflex is recommended for all trauma patients. Repeated clinical assessment is a mainstay of the diagnosis and ongoing management of brain injury from the hyper-acute phase onward, and any change in status or GCS score should be considered by the clinical team. Repeat assessments are done hourly or more frequently with any suspected change in status.

Once the patient has been assessed clinically and is stabilized vis-a-vis immediate life- or limb-threatening traumatic injury, additional diagnostic modalities are appropriate and necessary. Depending on competing polytrauma priorities (i.e., that may require urgent hemorrhage control), CT imaging is typically performed within 30 min of patient arrival to the hospital. Non-contrast head Computed Tomography (CT) is the workhorse of structural brain injury diagnosis because it has a high sensitivity and specificity for acute intracranial pathology, can be performed in minutes, and does not have common contraindications (such as contrast allergy or metal implants for a contrast-enhanced brain Magnetic Resonance Imaging (MRI)). These attributes are particularly relevant for the trauma setting, given the patient may not be alert, cooperative, or reliable, which can make obtaining a physical exam unreliable and an appropriate history of possible contraindications impossible, depending on the circumstances. Head CT, however, is less sensitive than brain MRI for detecting subtle traumatic brain injuries [22].

Blood-based biomarkers, glial fibrillary acidic protein (GFAP), ubiquitin carboxy-terminal hydrolase L1 (UCH-L1), and S100 calcium-binding protein (S100B) are increasingly being used to aid in the diagnosis of traumatic brain injury [16]. Some patients with negative head CT can still have persistent and debilitating symptoms due to cellular injury that is not structurally visible on head CT [16]. Blood-based biomarkers on the day of injury are also useful as adjuncts for severity of injury and predicting functional recovery at 6 months post-injury in trauma patients with GCS ≤ 12 [23,24,25]. At present, blood-based biomarkers do not play a role in the diagnosis of DoC in trauma patients, but they are increasingly relevant in the context of the new CBI-M framework for TBI classification (clinical assessments, biomarkers, imaging, and modifiers) [26].

The above summarizes the initial diagnosis and management of suspected TBI. An early diagnosis of DoC is tentative rather than relying on any one test independently, as there are many confounders to the clinical examination, including medication administration, acute intoxication, and post-ictal states, which can obscure the clinical evaluation and mimic disorders of consciousness [27]. Historically, a GCS score of ≤8 has been defined as coma, though this has been challenged by DoC literature as far back as 2002 for being overly simplified, and this definition has been redefined [5,28,29]. In an acute, severely injured trauma patient with impaired consciousness, however, GCS remains the gold standard for guiding assessment and management, and, along with the National Institutes of Health Stroke Scale, is the most commonly used [30,31]. More recent research in DoC suggests that the broad range of scores in each category of the GCS can be split up into more accurate and precise diagnostic categories first defined by the Aspen Consensus Criteria [29,32].

Following the diagnosis of a suspected acute DoC (i.e., best GCS < 13), trauma patients are admitted for close monitoring either to an intensive care unit (ICU) setting or stepdown level of care depending on the hospital system. The cornerstone of the hyper-acute management of these patients is repeat, serial GCS and pupillary assessments to promptly catch any signs of critical neuro worsening, which is defined in both the ACS and Seattle International Severe Traumatic Brain Injury Consensus Conference (SIBICC) guidelines and includes a decrease in the GCS motor score of 1 or more points, a decrease in pupillary reactivity, and other clinical signs [21,33]. Management otherwise hinges on carefully maintaining clinical parameters within normal physiologic ranges (i.e., oxygen saturation ≥ 94%, systolic blood pressure ≥ 110 mmHg, serum sodium 135–145 mEq/L, etc.) with the overarching goal of preventing additional secondary brain injury to the already-injured brain. A new addition to the ACS guidelines is the recommended use of prophylactic anti-epileptic medications for TBI patients who are at increased risk of early post-traumatic seizure, as seizures can exacerbate acute brain injury [16].

Patients with a best GCS < 8 or those with structural TBI anatomically suspected to be a high risk of developing elevated intracranial pressure (ICP) should have placement of an invasive ICP monitor within 4 h and active medical management to maintain ICP < 22 mmHg and cerebral perfusion pressure (CPP) 60–70 mmHg [21]. Recognizing and quickly treating ICP elevation is paramount to preventing the dreaded secondary brain damage. In the 2024 ACS guideline updates, a tiered approach to ICP management is provided, with specific interventions based on the severity of the condition [16].

### 2.2. Acute Window—24 h to Early Days of Hospitalization: Intensive Care

**Figure 2:**
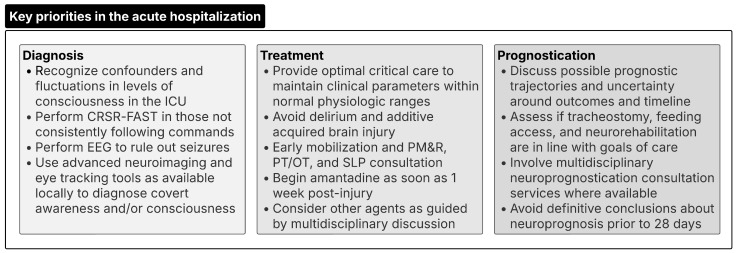
Key priorities in the acute hospitalization. This figure summarizes the key priorities for patients with suspected disorders of consciousness in during the acute hospitalization. ICU, intensive care unit; CRSR-FAST, Coma Recovery Scale-Revised For Accelerated Standardized Testing; EEG, electroencephalogram; PM&R, physical medicine and rehabilitation; PT/OT, physical therapy and occupational therapy; SLP, speech language pathology.

Much of the acute diagnosis, management, and prognostic anchoring for TBI/DoC occurs in the first week or two in the ICU. Patients with a decreased level of consciousness often require interventions that are restricted to ICU level of care, such as intubation with mechanical ventilation, hourly neurologic assessment, and carefully titrated blood pressure and ICP management, among others. Assessing DoC is especially challenging in the critical care environment, where the level of consciousness, a key factor in determining the patient’s clinical status as well as trajectory of recovery, is itself variable, and may furthermore be obscured by sedation, analgesia, and other ongoing confounders [5]. Acute disturbances in cognition and consciousness are widely prevalent in the ICU setting and have been associated with worse outcomes [34]. In truth, it can be near-impossible to reliably differentiate between the evolving natural history of DoC recovery after an acute brain injury and the appearance of a new acquired brain injury at this stage (e.g., delirium, septic encephalopathy, etc.) [35]. Surrogate decision makers are often placed in the situation of needing to make significant decisions about patient care (re: life and death, and invasive procedures), in discussion with the clinical teams, without knowing for certain what recovery state their loved one may ultimately achieve. In practice, this discussion often centers around the patient’s inability to reliably oxygenate and ventilate on their own, forcing an early decision about tracheostomy placement, typically within the first 7–10 days after injury [36,37]. The key priorities in the acute hospitalization are summarized in Figure 2.

#### 2.2.1. Early Acute (ICU Care): DoC Diagnosis

The diagnosis of DoC after acute traumatic injury incorporates clinical examination, behavioral phenotyping, neuroimaging, and electrophysiology, among other more emerging tests. Patients who sustain a brain injury associated with impairments in the cognitive functions of alertness and awareness can be said to have a DoC. The growing field of DoC has found that patients’ neurologic status traditionally described as “coma” likely spans several identifiable and differentiated states of consciousness, and that the use of GCS score 3–8 to define “coma” is an oversimplification of a broad spectrum of diagnostic range that can include both vegetative state (VS; or, more recently, unresponsive wakefulness syndrome [UWS]) and Minimally Conscious State (MCS). Lack of both alertness and awareness is a simplified DoC definition of coma. Patients in a VS/UWS recover alertness but continue to lack awareness [5,29]. MCS is defined as return of awareness and evidenced by various clinical signs including intact sleep–wake cycles and components of motor, verbal, or auditory function. MCS is subcategorized as MCS without language function (MCS−) and MCS with language function (MCS+) [5]. Emergence from minimally conscious state (eMCS) is defined as reliable yes–no communication and/or functional object use.

Because of the variability inherent in DoC and the confounders present, serial re-assessment of both diagnosis and prognosis is also required [38]. Confounding factors throughout the clinical course which may lead to underestimating a patient’s true level of conscious include central neurological deficits (e.g., aphasia, weakness), peripheral nerve injury, physical limitation, or an examiner’s subjective interpretation of ambiguous responses in addition to the confounders that carry over from the hyper-acute phase [39]. Additionally, fluctuation between states of consciousness or ability to exhibit behavior, a hallmark of DoC, can occur over minutes, hours, or days. To add to this, assessment measures for neurologic status and consciousness vary in time and resources necessary to yield a result, and, therefore, their utility may wax or wane as the patient goes through various stages of care, as well as expected fluctuation [40,41]. The importance of accurate, precise diagnosis in this environment—namely, capturing a trajectory of improvement—is critical as it can prevent premature withdrawal of life sustaining treatment (WLST) [42,43,44]. Unfortunately, in the ICU setting, routine bedside exams, such as the GCS or Full Outline of UnResponsiveness (FOUR) score, are insufficient to detect subtle signs of consciousness [28,45,46]. The original intention for developing the GCS was to characterize the severity of a TBI but not to diagnose a state of consciousness. The GCS remains widely used in the acute setting for its utility, but it provides an imprecise and antiquated assessment of level of consciousness.

While these traditional metrics can aid in identifying significant changes in clinical status and have the advantage of convenience, suspected DoC patients should also be assessed with standardized neurobehavioral assessment tools such as the Coma Recovery Scale-Revised (CRS-R) to more adequately diagnose and characterize DoC [5,46,47]. The bedside CRS-R examination does require specific knowledge and training, and takes significantly longer than a simple GCS, but after the first 48–72 h, most suspected TBI/DoC patients are sufficiently stabilized to allow this kind of detailed, specialized exam to be safely performed. As many as 40% of patients with DoC diagnosed as being in VS by GCS testing are actually in MCS following testing with the CRS-R; in other words, this group is frequently misdiagnosed by GCS testing—and is at high risk of potentially undergoing premature and goal-discordant WLST [6,48]. Clinical team consensus also underestimates the number of patients who emerge from MCS according to the CRS-R [6]. A streamlined version of the CRS-R called the Coma Recovery Scale-Revised For Accelerated Standardized Testing (CRS-R FAST) is now available and has been validated at a single site with substantial sensitivity, specificity, and accuracy comparable to the full CRS-R, and is undergoing additional validation in order to lower the threshold energy required to utilize such a standardized neurobehavioral assessment [49]. Other exam scales used are the Disability Rating Scale (DRS), Functional Independence Measure, and the Simplified Evaluation of Consciousness Disorders for timely assessment of DoC in the ICU [31].

Clinical assessment with the CRS-R or CRSR-FAST should be the cornerstone of DoC diagnosis in the ICU. Supplemental diagnostic modalities that may also take place in the acute setting include additional CT scans, MRI and functional MRI (fMRI), and electroencephalogram (EEG). CT is irreplicable for its efficiency in the acute setting, while MRI is more sensitive for subtle lesions [22]. Neuroimaging biomarkers are also under investigation for standard DoC and covert consciousness, including active, passive, and resting-state approaches. These include task-based fMRI, resting-state fMRI, EEG, and positron emission tomography (PET) [50,51].

EEG is commonly used in suspected DoC patients to rule out seizure, particularly status epilepticus which can mimic DoC, and specialized EEG has shown increasing diagnostic specificity for DoC [52]. Quantitative pupillometry is also a useful tool that provides more reliable and reproducible measurements than standard clinical assessment of pupillary reactivity but is not yet specifically used for diagnosing or ruling out DoC [16]. In addition, visual tracking or visual pursuit is a key element of the clinical examination of a patient with suspected DoC, and emerging advanced eye tracking tools may detect covert visual pursuit not readily apparent to the human eye and augment patient diagnostics based on eye tracking [53,54,55,56].

Cognitive-Motor Dissociation (CMD), covert consciousness, and covert awareness are overlapping conditions that are present in the acute setting. These terms globally refer to a patient who is aware and/or conscious, but this fact is not detectable on bedside clinical examination [48,57]. These phenomena have been identified in DoC patient populations with an incidence of 15–25%, and this incidence is higher after TBI-related DoC specifically [31,57]. In order to establish a diagnosis of CMD, functional imaging (e.g., fMRI) is used to monitor a patient’s brain activity while administering command prompts. Patients are found to have CMD if they cannot behaviorally exhibit command-following at the bedside but do have evidence of consistent brain activity on functional imaging that represents purposeful response. Although recovery outcomes for covertly aware/conscious patients are mixed across studies, establishing this diagnosis may allow additional communication with patients previously thought to be in a state of unconsciousness and allow clinical teams another means of respecting patient autonomy and providing goal-concordant care [31,39,57]. Specifically, this may open opportunities to include a patient who is behaviorally unresponsive in their management decisions or discussions of WLST in the future.

#### 2.2.2. Acute DoC Management

Once a DoC is suspected or diagnosed in the acute setting, DoC-specific management consists of several established and experimental pharmacological, neuro-modulatory (both non-invasive and invasive), and rehabilitative interventions [5]. Treatment of concomitant conditions (i.e., polytrauma, sepsis, delirium, other critical illness) is also central to DoC management in the ICU, as this aids to prevent worsening and better discern trajectories of recovery. The new ACS guidelines highlight the importance of early mobilization and the involvement of physical medicine and rehabilitation consultation, along with physical therapy, occupational therapy, and speech therapy in the initial day(s) after TBI/DoC [16]. Physical and occupational therapy can be missed in a DoC patient with decreased interactivity, but is an important adjunct to recovery in both the acute and post-acute setting. Multimodal sensory stimulation, including tactile, auditory, and vestibular sensory input are also low-risk, low-cost investments to support recovery [58].

Broadly, the current therapies for recovery can be broken down into several classes: pharmacologic, electromagnetic, mechanical, sensory, and regenerative. Pharmacological therapies with possible effectiveness for acute DoC include dopaminergic agents (amantadine, modafinil, and others), GABAergic agents (e.g., zolpidem, baclofen), antidepressants, antiseizure medications (as previously mentioned), orexin agonists, and possibly statins [16,38,59,60,61,62].

- Amantadine is the most well established and has been shown specifically to accelerate functional recovery as well as reduce disability early in recovery [38,60,63]. The ACS guidelines recommend amantadine (100–200 mg twice daily) be administered during the acute hospitalization for TBI/DoC as soon as one week after injury and after ruling out the confounding factors discussed earlier in this review [16].
- Modafinil, at a dose of 100–200 mg daily and started as soon as one-week post-injury, has also been studied extensively and is associated with an improved GCS score during treatment [63].
- Zolpidem, dosed at 10 mg daily in patients with prolonged DoC, has been shown to have a temporary paradoxical effect compared to its traditional clinical use in which it can sometimes awaken patients [62].
- Bromocriptine, levodopa/carbidopa, and other off-label dopaminergic modulators show some results in small studies and case reports [64].

Delirium affects up to 60% of critically ill patients with TBI and is associated with worse outcomes [65,66]. Medications such as sedating agents and analgesics, hospital room environment, and individual patient factors like age, pre-injury level of activity, and baseline comorbidities including pre-existing neuro-cognitive disease should be considered to minimize the risk of developing delirium, while maintaining patient comfort and safety (e.g., preventing self-extubation) [34].

Management of patients with DoC in the ICU is also closely tied with prognostication, and discussions with the surrogate(s) drive daily ICU decision-making and future planning. Perhaps the most tangible example of this inflection point in management is the decision to proceed with tracheostomy placement, as most acute DoC patients require mechanical ventilation due to decreased level of consciousness and an inability to protect their own airway. This is an extremely complex but seemingly time-pressured decision for both medical personnel and patient decision-makers. Because of the uncertainty surrounding long-term outcomes for patients with DoC and the simultaneous benefits of early tracheostomy placement (i.e., patient comfort, lower risk of ventilator-associated pneumonia), discussions should be thoughtful, thorough, and take the approach of presenting the best, worst, and most-likely outcomes for the patient based on their clinical status and the best available evidence. These decisions will often require multiple teams of physicians, including critical care, neurology, often a surgical sub-specialty, and palliative care.

#### 2.2.3. Acute DoC Prognosis

Some trauma patients with a severe, acute DoC do not survive their initial resuscitation, or progress rapidly to brain death, negating the need for prognostication [67]. These cases aside, for the vast majority of trauma patients with an acute DoC, even those deemed to have a “devastating neurologic injury”, prognostication during the hyper-acute time period (i.e., first 24 h) is both pre-mature and unreliable [68]. The presence of bilateral pupillary nonreactivity on admission is one of the few moderately reliable predictors of 6-month functional outcomes or in-hospital mortality, but 6-month functional outcomes do not capture long-term patient trajectories [68,69,70]. Prognostication should typically be deferred until the patient has been stabilized and at least 72 h of post-injury critical care has been provided [68]. Family discussions that acknowledge prognostic trajectories are necessary in the hyper-acute phase for patient surrogates to be informed about their global clinical status, but it should be emphasized that the natural course of DoC is not yet well understood and that possible outcomes are uncertain, and lie on a broad spectrum of recovery and functional status trajectories. Providers can help provide as much information about the possible range of outcomes expected to frame follow-up conversations with family or patient surrogates that may occur during this early “window of opportunity” to withdraw care, but the variability and unpredictability of prognosis means that providers often have very low certainty during this time, which makes comprehension and decision-making exponentially more difficult for surrogates undergoing stress [71]. Therefore, while it may be helpful to acknowledge decision points in the coming days (e.g., whether or not to proceed with tracheostomy, feeding tube placement, and aggressive neurorehabilitation), time and effort in the first 24 h is better spent on whole-patient management [19,71].

Prognostication in the acute phase includes discussing possible long-term outcomes, using predictive models, communication with families, concrete level-of-care decisions (e.g., tracheostomy, do-not-resuscitate status, WLST, etc.), and ethical considerations [5,38]. Unless the patient’s injury has caused irreparable damage to the brain stem, or unless the patient has documented predetermined wishes antithetical to required medical care, it is recommended that several days, or if possible weeks, of critical care and medical support be provided prior to concrete neuroprognostication [68]. During this time, serial assessment remains paramount, treatment of concomitant injuries should occur, and potential changes in trajectory should be noted and communicated to the patient’s surrogate(s). Consciousness improves rapidly in many, yet a substantial proportion of patients who may or may not have eventually recovered from their DoC (even within days to weeks) die instead due to WLST in that early time period.

Prognostication is best assessed with an assembly of clinical teams all in communication, assessment of the patient’s clinical status including concomitant injuries and comorbidities, and their current neurologic status using comprehensive, standardized, DoC-specific, behavioral assessment [39]. However, even with physician consensus, DoC is often misdiagnosed, which presumably affects prognostication and clinical course [6]. This only further emphasizes the need for standardized evaluation, such as the CRS-R, to prevent premature WLST and inform additional care decisions as well as research. As the possibility for eventual recovery becomes more apparent from ongoing research, clinical practice and guidelines about prognostication must continue to be updated. Emerging specialty neuroprognostication consultation services may help to better integrate multimodal outcome prediction and support this difficult decision-making process [72].

Even after two weeks, prognosis may still be uncertain, with some guidelines suggesting avoiding definitive conclusions about neuroprognosis prior to 28 days post-injury [43,68]. This is because recovery of consciousness and further recovery of independence/quality of life after brain injury from trauma can take months to years [43,73]. Prognostication should acknowledge substantial uncertainty due to the nature of the pathology, and, while physician experience is valuable, prognosis should not be based on personal anecdotal experience alone or incorporate narratives of nihilism [68].

Some factors do help us prognosticate, but they are imperfect. Younger age, male sex, and no evidence of intraventricular hemorrhage, intracranial mass effect, or subcortical contusion are some of the factors associated with better functional outcomes [12]. Older patients and those with higher injury severity scores have worse outcomes overall [74]. In addition to the externally-validated CRASH (Corticosteroid Randomization After Significant Head Injury) and IMPACT (International Mission for Prognosis and Analysis of Clinical Trials in TBI) prognostic models for TBI recovery, there is a TBI/DoC specific prognostic model for those admitted to inpatient DoC rehabilitation [30,75,76,77,78,79,80]. Nevertheless, all these prognostic models are imperfect and do not allow for certainty in the acute phase after traumatic injury. While recovery is better for patients with TBI than those with non-traumatic brain injuries, the timeline necessary for that recovery to take place may be longer [17,43]. Most patients experience improvement within the first year post-injury, but recovery can continue for five and even ten years after TBI/DoC [17]. One of the updates to the ACS guidelines for 2024 was the inclusion of comprehensive rehabilitation, which includes physical, cognitive, and psychological therapies, which may help improve this long-term recovery [16]. Prognostic uncertainty remains a key feature of recovery after acute brain injury and additional research is needed surrounding the natural history, outcomes and prognosis of DoC.

Recovery continues beyond the acute hospitalization, whether in post-acute rehabilitation settings or at home for those without funding (a sizable minority of TBI/DoC patients). While outside the scope of this review, post-acute DoC recovery is another rapidly growing area of investigation. DoC recovery trajectory and best possible prognosis prediction inform acute and sub-acute rehabilitation treatment decisions. Recent DoC literature has suggested delayed WLST as a compromise between having to make decisions about life sustaining treatment early in the hospitalization while still allowing the possibility of WLST if the patient does not achieve what they or their surrogate would deem “meaningful recovery” after several weeks [43].

## 3. Future Directions

TBI/DoC is increasingly viewed as both an acute condition and, for some, a chronic disease with long-term consequences that require ongoing follow-up and management [16]. We do need better tools for diagnosing and treating TBI/DoC in the acute setting. The lack of a good translational model for DoC limits mechanistic research, but advanced neuroimaging may soon fill that gap [81,82]. Investigation of potential treatments is primarily clinical and falls into several categories: pharmacologic, electromagnetic, mechanical, sensory, and regenerative treatments. This multifaceted approach will continue to be the focus of management-related research, while diagnosis, pathophysiology, and prognostication are simultaneously investigated [17,83].

Overall results of therapeutic interventions for DoC are mixed, but many promising trials are ongoing [84]. Common targets of treatment are the ascending arousal network, the default mode network, the frontoparietal/executive control network, and the thalamocortical network [83]. As discussed previously, the heterogeneity leading to DoC has made treatment and research into potential treatments much more complex [85].

Multiple electromagnetic treatment modalities are under investigation, including non-invasive brain stimulation to enhance arousal and alertness, deep brain stimulation (which is more invasive), transcranial magnetic stimulation (TMS) particularly over the M1 region, transcranial electric current stimulation, and peripheral nerve stimulation (such as trigeminal or median nerve stimulation) [64,84,85,86,87]. Neuro/gliogenesis via various mechanisms such as inducing proliferation of endogenous neural stem cells as well as administration of exogenous stem cells has been shown to restore synaptic connectivity and improve functional outcomes in animal models [88].

In terms of better diagnosing DoC states in patients after acute traumatic injury, multisite validation of the CRSR-FAST in the ICU setting is ongoing [32,89]. Other diagnostic modalities on the horizon include TMS-EEG, functional near-infrared spectroscopy (fNIRS), and anesthetic perturbation [90,91]. TMS has been shown to induce unique oscillatory rhythms on EEG in patients with DoC when compared to controls, which may also aid in detecting covert awareness in the acute ICU setting [90]. Anesthetic perturbation with propofol has been shown to generate a predictive index discernable on EEG (known as the adaptive reconfiguration index, ARI), which may help predict recovery of consciousness among DoC patients [92]. Additional validation of this finding in the acute TBI/DoC patient population is underway.

## 4. Conclusions

Disorders of consciousness after traumatic injury are a complex and evolving area of medicine. Standards of care from recent updates should include expeditious treatment of concomitant injuries, elimination of the possibility of DoC exam confounders, serial and standardized DoC-targeted neurological exams, and communication of the uncertainty of prognosis when making decisions regarding goals of care—and postponement of these decisions when prognosis is uncertain. Injury heterogeneity, lack of effective acute DoC treatments, and difficulty with accurate prognostication all contribute to the complexity of the field. Increased awareness of DoC among acute ICU providers and collaboration with the post-acute rehabilitation community should lead to better longitudinal studies, both mechanistic and therapeutic.

## Data Availability

No new data were created or analyzed in this study. Data sharing is not applicable to this article. This narrative review article included a review of 3486 articles that result from the Boolean search “(“brain injuries, traumatic” [MeSH Terms] OR “trauma”) AND (“Consciousness Disorders” [MeSH Terms] OR “Consciousness” [MeSH Terms])” on the PubMed database, among other notable articles and guidelines added for comprehensiveness.

## Abbreviations

The following abbreviations are used in this manuscript:

ACS	American College of Surgeons
ATLS	Advanced Trauma Life Support
CMD	Cognitive-motor dissociation
CPP	Cerebral perfusion pressure
CRASH	Corticosteroid Randomization After Significant Head Injury
CRS-R	Coma Recovery Scale-Revised
CRS-R FAST	Coma Recovery Scale-Revised for Accelerated Standardized Testing
CT	Computed tomography
DOC	Disorders(s) of consciousness
DRS	Disability Rating Scale
EEG	Electroencephalogram
eMCS	Emergence from minimally conscious state
fMRI	Functional MRI
FOUR	Full Outline of UnResponsiveness
GCS	Glasgow Coma Scale
GFAP	Glial fibrillary acidic protein
ICP	Intracranial pressure
ICU	Intensive care unit
IMPACT	International Mission for Prognosis and Analysis of Clinical Trials in TBI
MCS	Minimally conscious state
MRI	Magnetic resonance imaging
PET	Positron emission tomography
S100B	S100 calcium-binding protein
SIBICC	Seattle International Severe Traumatic Brain Injury Consensus Conference
TBI	Traumatic brain injury
TMS	Transcranial magnetic stimulation
UCH-L1	Ubiquitin carboxy-terminal hydrolase L1
UWS	Unresponsive wakefulness syndrome
VS	Vegetative state
WLST	Withdrawal of life sustaining treatment

## References

[1] Fogarty A.E., Guay C.S., Simoneau G., Colorado B.S., Segal G.R., Werner J.K., Ellenbogen J.M. (2019). Head Motion Predicts Transient Loss of Consciousness in Human Head Trauma: A Case-Control Study of Mixed Martial Artists. Am. J. Phys. Med. Rehabil..

[2] de Freitas Cardoso M.G., Faleiro R.M., de Paula J.J., Kummer A., Caramelli P., Teixeira A.L., de Souza L.C., Miranda A.S. (2019). Cognitive Impairment Following Acute Mild Traumatic Brain Injury. Front. Neurol..

[3] Bedard M., Taler V., Steffener J. (2018). Long-term prospective memory impairment following mild traumatic brain injury with loss of consciousness: Findings from the Canadian Longitudinal Study on Aging. Clin. Neuropsychol..

[4] Zare M.A., Ahmadi K., Zadegan S.A., Farsi D., Rahimi-Movaghar V. (2013). Effects of brain contusion on mild traumatic brain-injured patients. Int. J. Neurosci..

[5] Bodien Y.G., Busl K.M., Chang C.W.J., Claassen J., Gaspard N., Gosseries O., Helbok R., Massimini M., Naccache L., Newcombe V. (2026). Disorders of consciousness diagnosis, interventions, and prognostication for the intensivist: Report of the 2025 ISICEM roundtable. Intensive Care Med..

[6] Schnakers C., Vanhaudenhuyse A., Giacino J., Ventura M., Boly M., Majerus S., Moonen G., Laureys S. (2009). Diagnostic accuracy of the vegetative and minimally conscious state: Clinical consensus versus standardized neurobehavioral assessment. BMC Neurol..

[7] Korley F.K., Kelen G.D., Jones C.M., Diaz-Arrastia R. (2016). Emergency Department Evaluation of Traumatic Brain Injury in the United States, 2009–2010. J. Head Trauma Rehabil..

[8] Maas A.I.R., Menon D.K., Manley G.T., Abrams M., Akerlund C., Andelic N., Aries M., Bashford T., Bell M.J., Bodien Y.G. (2022). Traumatic brain injury: Progress and challenges in prevention, clinical care, and research. Lancet Neurol..

[9] Metting Z., Rodiger L.A., Regtien J.G., van der Naalt J. (2009). Delayed coma in head injury: Consider cerebral fat embolism. Clin. Neurol. Neurosurg..

[10] Singh A., Davis A.P., Taylor B., Peters S., Tirschwell D., Longstreth W.T., Mossa-Basha M., Nash M., Khot S.P. (2022). Cerebral fat embolism syndrome at a single trauma center. J. Stroke Cerebrovasc. Dis..

[11] Taylor C.A., Bell J.M., Breiding M.J., Xu L. (2017). Traumatic Brain Injury-Related Emergency Department Visits, Hospitalizations, and Deaths - United States, 2007 and 2013. MMWR Surveill. Summ..

[12] Kowalski R.G., Hammond F.M., Weintraub A.H., Nakase-Richardson R., Zafonte R.D., Whyte J., Giacino J.T. (2021). Recovery of Consciousness and Functional Outcome in Moderate and Severe Traumatic Brain Injury. JAMA Neurol..

[13] Seamon M.J., Feather C., Smith B.P., Kulp H., Gaughan J.P., Goldberg A.J. (2010). Just one drop: The significance of a single hypotensive blood pressure reading during trauma resuscitations. J. Trauma Acute Care Surg..

[14] Heppekcan D., Ekin S., Civi M., Aydin Tok D. (2019). Impact of Secondary Insults in Brain Death After Traumatic Brain Injury. Transpl. Transplant. Proc..

[15] American College of Surgeons ACS TQIP Best Practices in the Management of Traumatic Brain Injury. https://www.east.org/content/documents/45_podcast_acstqip_tbi_guidelines_2.pdf.

[16] American College of Surgeons Best Practices Guidelines for the Management of Traumatic Brain Injury. https://www.facs.org/media/vgfgjpfk/best-practices-guidelines-traumatic-brain-injury.pdf.

[17] Johnson-Black P.H., Carlson J.M., Vespa P.M. (2025). Traumatic brain injury and disorders of consciousness. Handb. Clin. Neurol..

[18] Schucht J.E., Rakhit S., Smith M.C., Han J.H., Brown J.B., Grigorian A., Gondek S.P., Smith J.W., Patel M.B., Maiga A.W. (2025). Beyond Glasgow Coma Scale: Prehospital prediction of traumatic brain injury. Surgery.

[19] Gondek S., Schroeder M.E., Sarani B. (2017). Assessment and Resuscitation in Trauma Management. Surg. Clin. N. Am..

[20] Fischer D., Edlow B.L. (2024). Coma Prognostication After Acute Brain Injury: A Review. JAMA Neurol..

[21] American College of Surgeons, Committee on Trauma (2025). Advanced Trauma Life Support (ATLS): Student Course Manual.

[22] Dabas M.M., Alameri A.D., Mohamed N.M., Mahmood R., Kim D.H., Samreen M., Kim J.W., Shehryar A., Gyambrah S., Bedros A.W. (2024). Comparative Efficacy of MRI and CT in Traumatic Brain Injury: A Systematic Review. Cureus.

[23] Goyal A., Failla M.D., Niyonkuru C., Amin K., Fabio A., Berger R.P., Wagner A.K. (2013). S100b as a prognostic biomarker in outcome prediction for patients with severe traumatic brain injury. J. Neurotrauma.

[24] Korley F.K., Jain S., Sun X., Puccio A.M., Yue J.K., Gardner R.C., Wang K.K.W., Okonkwo D.O., Yuh E.L., Mukherjee P. (2022). Prognostic value of day-of-injury plasma GFAP and UCH-L1 concentrations for predicting functional recovery after traumatic brain injury in patients from the US TRACK-TBI cohort: An observational cohort study. Lancet Neurol..

[25] Helmrich I., Czeiter E., Amrein K., Buki A., Lingsma H.F., Menon D.K., Mondello S., Steyerberg E.W., von Steinbuchel N., Wang K.K.W. (2022). Incremental prognostic value of acute serum biomarkers for functional outcome after traumatic brain injury (CENTER-TBI): An observational cohort study. Lancet Neurol..

[26] Manley G.T., Dams-O’Connor K., Alosco M.L., Awwad H.O., Bazarian J.J., Bragge P., Corrigan J.D., Doperalski A., Ferguson A.R., Mac Donald C.L. (2025). A new characterisation of acute traumatic brain injury: The NIH-NINDS TBI Classification and Nomenclature Initiative. Lancet Neurol..

[27] Stenberg M., Koskinen L.D., Jonasson P., Levi R., Stalnacke B.M. (2017). Computed tomography and clinical outcome in patients with severe traumatic brain injury. Brain Inj..

[28] Bodien Y.G., Barra A., Temkin N.R., Barber J., Foreman B., Vassar M., Robertson C., Taylor S.R., Markowitz A.J., Manley G.T. (2021). Diagnosing Level of Consciousness: The Limits of the Glasgow Coma Scale Total Score. J. Neurotrauma.

[29] Giacino J.T., Ashwal S., Childs N., Cranford R., Jennett B., Katz D.I., Kelly J.P., Rosenberg J.H., Whyte J., Zafonte R.D. (2002). The minimally conscious state: Definition and diagnostic criteria. Neurology.

[30] Dijkland S.A., Foks K.A., Polinder S., Dippel D.W.J., Maas A.I.R., Lingsma H.F., Steyerberg E.W. (2020). Prognosis in Moderate and Severe Traumatic Brain Injury: A Systematic Review of Contemporary Models and Validation Studies. J. Neurotrauma.

[31] Murtaugh B., Olson D.M., Badjatia N., Lewis A., Aiyagari V., Sharma K., Creutzfeldt C.J., Falcone G.J., Shapiro-Rosenbaum A., Zink E.K. (2025). Caring for Coma after Severe Brain Injury: Clinical Practices and Challenges to Improve Outcomes: An Initiative by the Curing Coma Campaign. Neurocrit. Care.

[32] Weaver J.A., Cogan A.M., O’Brien K.A., Hansen P., Giacino J.T., Whyte J., Bender Pape T., van der Wees P., Mallinson T. (2022). Determining the Hierarchy of Coma Recovery Scale-Revised Rating Scale Categories and Alignment with Aspen Consensus Criteria for Patients with Brain Injury: A Rasch Analysis. J. Neurotrauma.

[33] Hawryluk G.W.J., Aguilera S., Buki A., Bulger E., Citerio G., Cooper D.J., Arrastia R.D., Diringer M., Figaji A., Gao G. (2019). A management algorithm for patients with intracranial pressure monitoring: The Seattle International Severe Traumatic Brain Injury Consensus Conference (SIBICC). Intensive Care Med..

[34] Delnero C., Berger K., O’Phelan K.H. (2025). Impaired Consciousness in the Traumatic Brain Injury Patient. Neurosurg. Clin. N. Am..

[35] Roberson S.W., Patel M.B., Dabrowski W., Ely E.W., Pakulski C., Kotfis K. (2021). Challenges of Delirium Management in Patients with Traumatic Brain Injury: From Pathophysiology to Clinical Practice. Curr. Neuropharmacol..

[36] Azari Jafari A., Mirmoeeni S., Momtaz D., Kotzur T., Murtha G., Garcia C., Moran M., Martinez P., Chen K., Krishnakumar H. (2024). Early Versus Late Tracheostomy in Patients with Traumatic Brain Injury: A US Nationwide Analysis. Neurocrit. Care.

[37] Krishnamoorthy V., Hough C.L., Vavilala M.S., Komisarow J., Chaikittisilpa N., Lele A.V., Raghunathan K., Creutzfeldt C.J. (2019). Tracheostomy After Severe Acute Brain Injury: Trends and Variability in the USA. Neurocrit. Care.

[38] Giacino J.T., Katz D.I., Schiff N.D., Whyte J., Ashman E.J., Ashwal S., Barbano R., Hammond F.M., Laureys S., Ling G.S.F. (2018). Practice guideline update recommendations summary: Disorders of consciousness: Report of the Guideline Development, Dissemination, and Implementation Subcommittee of the American Academy of Neurology; the American Congress of Rehabilitation Medicine; and the National Institute on Disability, Independent Living, and Rehabilitation Research. Neurology.

[39] Bodien Y.G., Fecchio M., Gilmore N., Freeman H.J., Sanders W.R., Meydan A., Lawrence P.K., Atalay A.S., Kirsch J., Healy B.C. (2026). Multimodal Biomarkers of Consciousness in Acute Severe Traumatic Brain Injury. J. Neurotrauma.

[40] Papadimitriou C., Weaver J.A., Guernon A., Walsh E., Mallinson T., Pape T.L.B. (2022). “Fluctuation is the norm”: Rehabilitation practitioner perspectives on ambiguity and uncertainty in their work with persons in disordered states of consciousness after traumatic brain injury. PLoS ONE.

[41] Barra A., Bodien Y.G., Tan C.O., Martens G., Malone C., Giacino J.T. (2025). Behavioral Fluctuation in Disorders of Consciousness: A Retrospective Analysis. Arch. Phys. Med. Rehabil..

[42] Sanders W.R., Barber J.K., Temkin N.R., Foreman B., Giacino J.T., Williamson T., Edlow B.L., Manley G.T., Bodien Y.G. (2024). Recovery Potential in Patients Who Died After Withdrawal of Life-Sustaining Treatment: A TRACK-TBI Propensity Score Analysis. J. Neurotrauma.

[43] Williams A., Bass G.D., Hampton S., Klinedinst R., Giacino J.T., Fischer D. (2025). Delayed Withdrawal of Life-Sustaining Treatment in Disorders of Consciousness: Practical and Theoretical Considerations. Neurocrit. Care.

[44] McCrea M.A., Giacino J.T., Barber J., Temkin N.R., Nelson L.D., Levin H.S., Dikmen S., Stein M., Bodien Y.G., Boase K. (2021). Functional Outcomes Over the First Year After Moderate to Severe Traumatic Brain Injury in the Prospective, Longitudinal TRACK-TBI Study. JAMA Neurol..

[45] Wijdicks E.F., Kramer A.A., Rohs T., Hanna S., Sadaka F., O’Brien J., Bible S., Dickess S.M., Foss M. (2015). Comparison of the Full Outline of UnResponsiveness score and the Glasgow Coma Scale in predicting mortality in critically ill patients. Crit. Care Med..

[46] Seel R.T., Sherer M., Whyte J., Katz D.I., Giacino J.T., Rosenbaum A.M., Hammond F.M., American Congress of Rehabilitation Medicine, Brain Injury-Interdisciplinary Special Interest Group, Disorders of Consciousness Task Force. (2010). Assessment scales for disorders of consciousness: Evidence-based recommendations for clinical practice and research. Arch. Phys. Med. Rehabil..

[47] Giacino J.T., Kalmar K., Whyte J. (2004). The JFK Coma Recovery Scale-Revised: Measurement characteristics and diagnostic utility. Arch. Phys. Med. Rehabil..

[48] Edlow B.L., Fins J.J. (2018). Assessment of Covert Consciousness in the Intensive Care Unit: Clinical and Ethical Considerations. J. Head Trauma Rehabil..

[49] Bodien Y.G., Vora I., Barra A., Chiang K., Chatelle C., Goostrey K., Martens G., Malone C., Mello J., Parlman K. (2023). Feasibility and Validity of the Coma Recovery Scale-Revised for Accelerated Standardized Testing: A Practical Assessment Tool for Detecting Consciousness in the Intensive Care Unit. Ann. Neurol..

[50] Wang J., Lai Q., Han J., Qin P., Wu H. (2024). Neuroimaging biomarkers for the diagnosis and prognosis of patients with disorders of consciousness. Brain Res..

[51] Xu L.B., Hampton S., Fischer D. (2024). Neuroimaging in Disorders of Consciousness and Recovery. Phys. Med. Rehabil. Clin. N. Am..

[52] Curley W.H., Bodien Y.G., Zhou D.W., Conte M.M., Foulkes A.S., Giacino J.T., Victor J.D., Schiff N.D., Edlow B.L. (2022). Electrophysiological correlates of thalamocortical function in acute severe traumatic brain injury. Cortex.

[53] Aklepi G., Manolovitz B., Robayo L.E., Sarafraz A., Blandino C.F., Arwari B., Sobczak E., Bass D., Ghamasaee P., Bolanos Saavedra A. (2024). Covert Tracking to Immersive Stimuli in Traumatic Brain Injury Subjects with Disorders of Consciousness. J. Neurotrauma.

[54] Alkhachroum A., Aklepi G., Sarafraz A., Robayo L.E., Manolovitz B.M., Blandino C.F., Arwari B., Sobczak E., Bass D.H., Ghamasaee P. (2023). Covert Tracking to Visual Stimuli in Comatose Patients with Traumatic Brain Injury. Neurology.

[55] Kondziella D., Bender A., Diserens K., van Erp W., Estraneo A., Formisano R., Laureys S., Naccache L., Ozturk S., Rohaut B. (2020). European Academy of Neurology guideline on the diagnosis of coma and other disorders of consciousness. Eur. J. Neurol..

[56] Zurek G., Binder M., Kunka B., Kosikowski R., Rodzen M., Karas D., Mucha G., Olejniczak R., Goraczko A., Kujawa K. (2024). Can Eye Tracking Help Assess the State of Consciousness in Non-Verbal Brain Injury Patients?. J. Clin. Med..

[57] Bodien Y.G., Allanson J., Cardone P., Bonhomme A., Carmona J., Chatelle C., Chennu S., Conte M., Dehaene S., Finoia P. (2024). Cognitive Motor Dissociation in Disorders of Consciousness. N. Engl. J. Med..

[58] Weaver J.A., Watters K., Cogan A.M. (2023). Interventions Facilitating Recovery of Consciousness Following Traumatic Brain Injury: A Systematic Review. Occup. Ther. J. Res..

[59] Girard Pepin R., Seyfzadeh F., Williamson D., Gosseries O., Duclos C. (2025). Pharmacological therapies for early and long-term recovery in disorders of consciousness: Current knowledge and promising avenues. Expert Rev. Neurother..

[60] Giacino J.T., Whyte J., Bagiella E., Kalmar K., Childs N., Khademi A., Eifert B., Long D., Katz D.I., Cho S. (2012). Placebo-controlled trial of amantadine for severe traumatic brain injury. N. Engl. J. Med..

[61] Tang H., Zhu Q., Li W., Qin S., Gong Y., Wang H., Shioda S., Li S., Huang J., Liu B. (2019). Neurophysiology and Treatment of Disorders of Consciousness Induced by Traumatic Brain Injury: Orexin Signaling as a Potential Therapeutic Target. Curr. Pharm. Des..

[62] Du B., Shan A., Zhang Y., Zhong X., Chen D., Cai K. (2014). Zolpidem arouses patients in vegetative state after brain injury: Quantitative evaluation and indications. Am. J. Med. Sci..

[63] Seifi A., Hassannezhad S., Mosaddeghi-Heris R., Haji Kamanaj Olia A., Adib A., Hafeez S., Barthol C. (2023). Consciousness Recovery in Traumatic Brain Injury: A Systematic Review Comparing Modafinil and Amantadine. Clin. Neuropharmacol..

[64] Meyer M.J., Megyesi J., Meythaler J., Murie-Fernandez M., Aubut J.A., Foley N., Salter K., Bayley M., Marshall S., Teasell R. (2010). Acute management of acquired brain injury Part III: An evidence-based review of interventions used to promote arousal from coma. Brain Inj..

[65] Hughes C.G., Patel M.B., Jackson J.C., Girard T.D., Geevarghese S.K., Norman B.C., Thompson J.L., Chandrasekhar R., Brummel N.E., May A.K. (2017). Surgery and Anesthesia Exposure Is Not a Risk Factor for Cognitive Impairment After Major Noncardiac Surgery and Critical Illness. Ann. Surg..

[66] Mart M.F., Thompson J.L., Ely E.W., Pandharipande P.P., Patel M.B., Wilson J.E., Williams Roberson S., Birdrow C.I., Raman R., Brummel N.E. (2022). In-Hospital Depressed Level of Consciousness and Long-Term Functional Outcomes in ICU Survivors. Crit. Care Med..

[67] Greer D.M., Shemie S.D., Lewis A., Torrance S., Varelas P., Goldenberg F.D., Bernat J.L., Souter M., Topcuoglu M.A., Alexandrov A.W. (2020). Determination of Brain Death/Death by Neurologic Criteria: The World Brain Death Project. JAMA.

[68] Muehlschlegel S., Rajajee V., Wartenberg K.E., Alexander S.A., Busl K.M., Creutzfeldt C.J., Fontaine G.V., Hocker S.E., Hwang D.Y., Kim K.S. (2024). Guidelines for Neuroprognostication in Critically Ill Adults with Moderate-Severe Traumatic Brain Injury. Neurocrit. Care.

[69] Russell M.E., Ivanhoe C.B., Reed E.A. (2024). Prognostication and Trajectories of Recovery in Disorders of Consciousness. Phys. Med. Rehabil. Clin. N. Am..

[70] Hammond F.M., Giacino J.T., Nakase Richardson R., Sherer M., Zafonte R.D., Whyte J., Arciniegas D.B., Tang X. (2019). Disorders of Consciousness due to Traumatic Brain Injury: Functional Status Ten Years Post-Injury. J. Neurotrauma.

[71] Kitzinger J., Kitzinger C. (2013). The ‘window of opportunity’ for death after severe brain injury: Family experiences. Sociol. Health Illn..

[72] Fischer D., Reyes-Esteves S., Law C., Ford A., Schwab P., Abella B.S., Schneider A.L.C., Kumar M.A. (2025). Implementation of a specialized neuroprognostication consultation program and associated provider attitudes: A survey-based study. Resusc. Plus.

[73] Swarna S., Saadon J.R., Robertson J., Vagal V., Cleri N.A., Butler K., Cheng X., Hua Y., Aghili S.M.M., Uwakwe C. (2026). Fast and Slow Recovery of Consciousness Following Traumatic Brain Injury. Neurocrit. Care.

[74] Cassinat J., Nygaard J., Hoggard C., Hoffmann M. (2024). Predictors of mortality and rehabilitation location in adults with prolonged coma following traumatic brain injury. PM R.

[75] Collaborators M.C.T., Perel P., Arango M., Clayton T., Edwards P., Komolafe E., Poccock S., Roberts I., Shakur H., Steyerberg E. (2008). Predicting outcome after traumatic brain injury: Practical prognostic models based on large cohort of international patients. BMJ.

[76] Steyerberg E.W., Mushkudiani N., Perel P., Butcher I., Lu J., McHugh G.S., Murray G.D., Marmarou A., Roberts I., Habbema J.D. (2008). Predicting outcome after traumatic brain injury: Development and international validation of prognostic scores based on admission characteristics. PLoS Med..

[77] Perel P., Edwards P., Wentz R., Roberts I. (2006). Systematic review of prognostic models in traumatic brain injury. BMC Med. Inf. Inform. Decis. Mak..

[78] Han J., King N.K., Neilson S.J., Gandhi M.P., Ng I. (2014). External validation of the CRASH and IMPACT prognostic models in severe traumatic brain injury. J. Neurotrauma.

[79] Snider S.B., Temkin N.R., Barber J., Edlow B.L., Giacino J.T., Hammond F.M., Izzy S., Kowalski R.G., Markowitz A.J., Rovito C.A. (2023). Predicting Functional Dependency in Patients with Disorders of Consciousness: A TBI-Model Systems and TRACK-TBI Study. Ann. Neurol..

[80] Panczykowski D.M., Puccio A.M., Scruggs B.J., Bauer J.S., Hricik A.J., Beers S.R., Okonkwo D.O. (2012). Prospective independent validation of IMPACT modeling as a prognostic tool in severe traumatic brain injury. J. Neurotrauma.

[81] Edlow B.L., Claassen J., Schiff N.D., Greer D.M. (2021). Recovery from disorders of consciousness: Mechanisms, prognosis and emerging therapies. Nat. Rev. Neurol..

[82] Snider S.B., Edlow B.L. (2020). MRI in disorders of consciousness. Curr. Opin. Neurol..

[83] Edlow B.L., Sanz L.R.D., Polizzotto L., Pouratian N., Rolston J.D., Snider S.B., Thibaut A., Stevens R.D., Gosseries O., Curing Coma C. (2021). Therapies to Restore Consciousness in Patients with Severe Brain Injuries: A Gap Analysis and Future Directions. Neurocrit. Care.

[84] De Koninck B.P., Brazeau D., Deshaies A.A., Briand M.M., Maschke C., Williams V., Arbour C., Williamson D., Duclos C., Bernard F. (2024). Modulation of brain activity in brain-injured patients with a disorder of consciousness in intensive care with repeated 10-Hz transcranial alternating current stimulation (tACS): A randomised controlled trial protocol. BMJ Open.

[85] Sharma N., Chahal A., Rai R.H., Wojcik B.M., Alfaifi B.J., Vajrala K.R., Sidiq M., Sharma A. (2025). Effects of non-invasive brain stimulation on arousal and alertness among traumatic brain injury patients with disorders of consciousness or persistent vegetative State: A systematic review. Acta Neurol. Belg..

[86] Shen L., Huang Y., Liao Y., Yin X., Huang Y., Ou J., Ouyang H., Chen Z., Long J. (2023). Effect of high-frequency repetitive transcranial magnetic stimulation over M1 for consciousness recovery after traumatic brain injury. Brain Behav..

[87] Kundu B., Brock A.A., Englot D.J., Butson C.R., Rolston J.D. (2018). Deep brain stimulation for the treatment of disorders of consciousness and cognition in traumatic brain injury patients: A review. Neurosurg. Focus.

[88] Ichim T.E., Ramos R.A., Rath A., Castellano J., Azimi N., Veltmeyer J.D., Koumjian M., Ma N.E., Bajnath A., Lin E. (2025). Reversing coma by senolytics and stem cells: The future is now. J. Transl. Med..

[89] Weaver J.A., Cogan A.M., Kozlowski A.J., Grady-Dominguez P., O’Brien K.A., Bodien Y.G., Graham J., Aichele S., Ford P., Kot T. (2024). Interpreting Change in Disorders of Consciousness Using the Coma Recovery Scale-Revised. J. Neurotrauma.

[90] Formaggio E., Cavinato M., Storti S.F., Tonin P., Piccione F., Manganotti P. (2016). Assessment of Event-Related EEG Power After Single-Pulse TMS in Unresponsive Wakefulness Syndrome and Minimally Conscious State Patients. Brain Topogr..

[91] Abdalmalak A., Milej D., Norton L., Debicki D.B., Owen A.M., Lawrence K.S. (2021). The Potential Role of fNIRS in Evaluating Levels of Consciousness. Front. Hum. Neurosci..

[92] Girard Pepin R., Jutras R., Lahaie L., Bazregarzadeh H., Martin A., Maschke C., Badawy M., Letourneau J., Plourde G., Briand M.M. (2026). Effects of propofol on high-density EEG in disorders of consciousness: An exploratory study of patient-specific neurophysiological modulation. Neuroimage.

